# Long-term changes in wearable sensor data in people with and without Long Covid

**DOI:** 10.1038/s41746-024-01238-x

**Published:** 2024-09-13

**Authors:** Jennifer M. Radin, Julia Moore Vogel, Felipe Delgado, Erin Coughlin, Matteo Gadaleta, Jay A. Pandit, Steven R. Steinhubl

**Affiliations:** 1 Scripps Research Translational Institute, La Jolla, CA 92037 USA; 2 Purdue University, West Lafayette, IN 47907 USA

**Keywords:** Viral infection, Epidemiology

## Abstract

To better understand the impact of Long COVID on an individual, we explored changes in daily wearable data (step count, resting heart rate (RHR), and sleep quantity) for up to one year in individuals relative to their pre-infection baseline among 279 people with and 274 without long COVID. Participants with Long COVID, defined as symptoms lasting for 30 days or longer, following a SARS-CoV-2 infection had significantly different RHR and activity trajectories than those who did not report Long COVID and were also more likely to be women, younger, unvaccinated, and report more acute-phase (first 2 weeks) symptoms than those without Long COVID. Demographic, vaccine, and acute-phase sensor data differences could be used for early identification of individuals most likely to develop Long COVID complications and track objective evidence of the therapeutic efficacy of any interventions.

Trial Registration: https://classic.clinicaltrials.gov/ct2/show/NCT04336020.

An estimated 7.5% to 41% of nonhospitalized people who are infected with SARS-COV-2 go on to develop Long COVID^1^, which in 2023 was defined by the CDC as symptoms lasting for 4 weeks or longer after initial infection^2^. Among those who develop post-COVID syndromes and go on to seek treatment, it is estimated that more than half have moderately severe or greater functional limitations and 20% are unable to work^3^. Despite the high disease burden of Long COVID, very little is known about the natural history of this multi-system disease, its impact on affected individual’s physiology and behaviors (i.e., activity and sleep quantity), and risk factors for both ongoing symptoms and different manifestations. Longitudinal wearable sensor data allows for a person’s long-term changes following an acute COVID infection to be compared to their unique, pre-infection baseline data, controlling for the wide range of inter-individual variability in these biometrics.

Combining objective measures of changes in physiology and behavior and the presence and type of persistent symptoms in individuals who experienced an acute COVID infection may be useful for identifying people who are more likely to experience long-term COVID sequelae and potentially provide insights into individual pathophysiologic mechanisms that can guide therapeutic interventions and track their efficacy. The aim of this study was to evaluate if participants who experienced ongoing symptoms had different sensor deviation patterns and trajectories or other clinical differences compared to individuals who tested positive for COVID-19 but whose symptoms resolved.

A total of 595 participants with a prior documented COVID infection responded to the survey out of 2410 who were sent the survey. After excluding duplicate surveys (only the first survey filled out was used), those with missing symptom onset dates, or symptom onset dates that were >7 days before or after a test result, or those who reported a symptom end date before symptom onset date, we were left with 553 participants - 279 with long COVID and 274 without Long COVID.

Of the 553 respondents, 176 (96.3%) reported receiving outpatient care and 5 (2.8%) reported hospitalization during their acute infection. Thirty-eight out of 39 (97.4%) participants who reported not receiving any COVID-19 vaccine before testing positive had a symptom onset between January 1, 2020- November 29, 2021.

As noted in Table 1, several key differences were found in the characteristics of participants with Long COVID compared to those without. Of note, women and younger participants (<65 years old) were more likely to report Long COVID. Among participants with Long COVID, 30 (10.8%) reported their symptoms during the acute phase as being severe or very severe compared to 8 (2.9%) among those without Long COVID.

**Table 1:** Characteristics of participants by those with and without Long COVID, *n* = 553

Long COVID	Yes	No	*p*-value
*n* (%)	279 (50.5)	274 (49.67)	
Gender			0.00494
Female	196 (72.9)	163 (59.7)	
Male	71 (26.4)	108 (39.6)	
Other	2 (0.7)	2 (0.7)	
Age group			0.02
18–40	55 (20.5)	51 (18.7)	
41–64	142 (52.8)	119 (43.4)	
65+	72 (26.8)	103 (37.7)	
Severity			<0.0001
Mild	43 (15.4)	74 (27.0)	
Moderate	206 (73.8)	192 (70.1)	
Severe	30 (10.8)	8 (2.9)	
Condition			
ME/CFS	33 (11.8)	5 (1.8)	<0.0001
PEM	53 (19.0)	5 (1.8)	<0.0001
Myocarditis/pericarditis	8 (2.9)	3 (1.1)	0.14
POTS	20 (7.2)	8 (2.9)	0.023
Symptoms reported during acute phase			
Fever	116 (41.6)	91 (33.2)	0.04
Cough	201 (72.0)	181 (66.1)	0.13
Fatigue	176 (63.1)	137 (50.0)	0.0019
Body ache	138 (49.5)	99 (36.1)	0.0015
Headache	162 (57.1)	127 (46.4)	0.006
Sore throat	169 (60.6)	163 (59.5)	0.79
Diarrhea or vomiting	21 (14.7)	41 (7.7)	0.0088
Loss of smell or taste	27 (10.0)	17 (6.2)	0.10
Shortness of breath	42 (15.1)	16 (5.8)	0.0004
Congestion	205 (73.5)	199 (72.6)	0.82
Length of sick, days (mean, STD)	281.0 (251.0)^a^	12.7 (6.7)	<0.0001
Vaccinated >14 days prior to symptom onset			
First dose	243 (87.1)	271 (98.9)	<0.0001
Second dose	242 (86.7)	271 (98.9)	<0.0001
Symptom onset date			<0.0001
January 1, 2020–November 29, 2021	59 (21.2)	10 (3.7)	
November 30, 2021–March 31, 2022	68 (24.4)	35 (12.8)	
April 1, 2022–March 15, 2023	152 (54.5)	229 (83.6)	

^a^Number of sick days as of March 16, 2023, if <0 then length sick was deleted.

Symptom severity (mild = asymptomatic, very mild, mild); moderate = moderate; severe = severe or very severe); Myalgic Encephalomyelitis/Chronic Fatigue Syndrome (*ME/CFS*); Post-exertional malaise (*PEM*); Postural orthostatic tachycardia syndrome (*POTS*)

On average, individuals who reported Long COVID had resting heart rates (RHRs), which remained above their individual baseline (*z*-score average did not include 0) for 133 days whereas those without Long COVID returned to baseline after 71 days. Activity, as determined by change in daily step count relative to pre-infection, was significantly lower in those with Long COVID compared to those without during parts of the acute phase and later; however, on average those with Long COVID returned to baseline at 17 days and those without at 20 days post-symptom onset. There were no significant daily differences in sleep quantity between the two groups. On average those with Long COVID returned to baseline at 14 days, and those without at 9 days (Fig. 1).

**Fig. 1:**
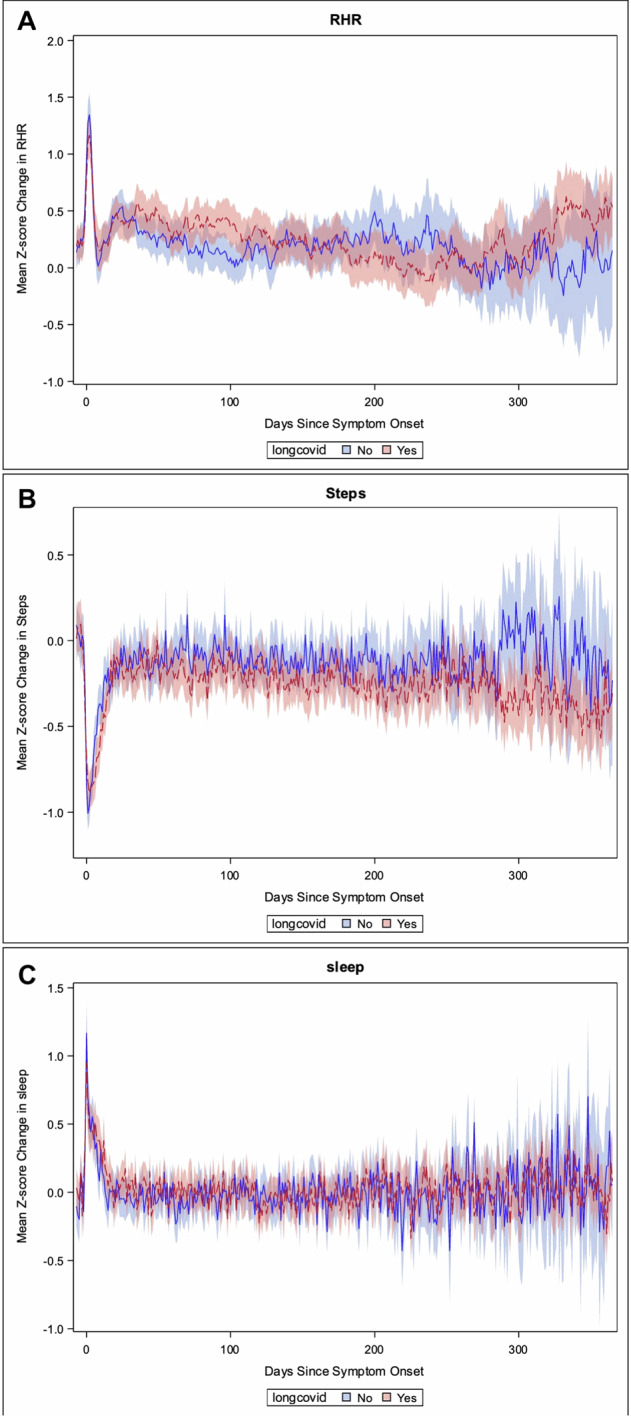
Comparison of daily mean z-scores (95% CI) from 7 days prior to 1 year after symptom onset between those who did and did not develop long COVID (yes/no). The *z*-score compares each indivdual to their pre-infectious baseline >7 days prior to symptom onset. **A** Resting heart rate (RHR), **B** step count, **C** sleep quantity. Out of 553 participatns, 409 provided step data, 365 sleep quantity, and 365 provided RHR. Only data from Fitbits were included in this figure.

Prior research from DETECT has shown that, on average, RHR stays elevated for 2–3 months after symptom onset from a COVID-19 infection, but some individuals had RHR deviations that lasted for up to 6 months and did not go back to their pre-infection baseline^4^, potentially as a result of the post-acute sequela of SARS-CoV-2 infection (PASC), or Long COVID. Here, we expand on those early findings with objective physiologic and behavioral data extending to 365 days and beyond following infection, and participant-reported symptoms and diagnoses for a mean of 11 months from the time of their acute COVID infection. The strength of our findings is that they provide objective evidence of prolonged physiologic derangement in individuals experiencing Long COVID relative to their healthy, pre-infection baseline. Individuals who did not report having Long COVID experienced significantly shorter, albeit still prolonged, physiologic deviations.

We found that individuals with Long COVID symptoms experience a significantly different trajectory in changes from their pre-infection RHR norms compared to those without Long COVID. Understanding the natural history of Long COVID by characterizing acute-phase changes and symptoms can potentially help identify individuals early on who have a greater likelihood of developing long-term symptoms or complications and identifying individuals who would have the greatest benefit from early interventions, such as rest^5^ or pharmaceutical interventions such as metformin which is most effective in preventing Long COVID if given within <3 days from symptom onset^6^.

We found that participants who were infected early during the pandemic or who were unvaccinated were more likely to report Long COVID. However, since the COVID-19 vaccine wasn’t available until later in the pandemic, it is hard to know whether the difference in duration and risk of Long COVID is a result of differences in early strains of the virus, from being vaccinated, or a combination. Other studies have found that vaccination reduces risk of developing Long COVID by 15 to 41%^7,8^.

This was a prospective observational study with ongoing enrollment; therefore, some participants have been in the study longer than others. All the survey data was self-reported and therefore it is possible that participants were not aware of their diagnoses. It is possible that participants who responded to our Long COVID survey were more likely to suffer from Long COVID, resulting in an oversampling of these cases. We also had small sample sizes which may have reduced our generalizability. It is also possible that participants with the greatest impairment may have been unable to complete our survey. Recall bias is also a possibility, especially for participants who were infected early in the pandemic. There was more drop-off in sensor data over time in the group who did not have Long COVID compared to the group who did, which may have biased our results (Supplementary Fig. 1). Finally, we did not address the possible seasonality effect on wearable data.

This is one of the longest prospective studies to continuously collect sensor data from participants who contracted COVID-19. Most importantly, data availability well preceded acute infection, providing objective evidence of an individual’s true pre-infection baseline. It is also the only one to collect both acute and long-term symptoms/conditions from participants to better understand how their acute-phase response may relate to the development of long-term conditions. Gaining a more complete picture of the natural history of Long COVID and how it evolves over time is possible through continuously collected smartwatch data. In the future, these findings could be used to provide individualized and early treatment to participants who are most likely to develop Long COVID conditions.

Our study provides objective evidence for a pathophysiological response among participants with Long COVID that has been described in other studies^9^. Long COVID can have debilitating impacts on function with a third of Long COVID cases unable to live at home without assistance^5^. Objectively collected data using smartwatches supplement patient’s lived experiences. We found that those with a lack of symptom resolution experienced overall greater declines in step counts, and different RHR patterns, and were more likely to experience systemic symptoms during the acute phase of their infection, were more likely to be women, younger, and less likely to be vaccinated.

## Methods

For this study, we included participants who had been prospectively enrolled in the DETECT study (DETECT.scripps.edu) between March 25, 2020 and March 16, 2023. Participants downloaded the MyDataHelps research app, available on Android and iOS^10^, and filled out an electronic consent form allowing them to share daily sensor data (RHR, activity, and sleep quantity) from their fitness tracker or smartwatch. Participants could also fill out ongoing surveys whenever they got sick (symptom onset date, symptoms), tested for COVID-19, or received a COVID-19 vaccination. For this assessment, we sent out a one-time 5-question survey to individuals who reported a COVID-19 positive test between January 19-March 7th, 2023 via email, SMS, and/or push notification to evaluate overall severity, symptom pattern, health care seeking, whether they developed any specific Long COVID-related sequelae, and recovery. We categorized an individual as having Long COVID if their symptoms lasted for 30 days or longer. For Table 1, chi-squared tests were used to compare differences in frequency among categorical variables and *t*-tests were used to compare differences in means between continuous variables.

The DETECT study prospectively and continuously collects COVID-19-positive participants’ physiologic and behavioral data via a wearable sensor both before and during the acute phase, and then for up to several years after their acute infection. This broader DETECT study is device-agnostic and can receive data from any sensor that is compatible with Google Fit or Apple HealthKit. In addition to sensor data, self-reported demographics, acute-phase symptoms, and vaccination data are collected prospectively.

### Data analysis

Participants had to report a symptom onset that was within 7 days before or after their COVID-19 positive test date. We compared the length of acute symptoms (date symptoms ended minus first symptom onset date that occurred 7 days before or after a COVID-19 positive test), frequency of acute-phase symptoms, age, gender, wave of COVID-19 when first diagnosed, and prior vaccination across participants with and without Long COVID. Participants were considered vaccinated with one or two doses if it had been >14 days before symptom onset. When calculating the length of chronic symptoms for participants with Long COVID symptoms, the length from symptom onset until March 16, 2023 was used.

For the figure, we only used sensor data from Fitbit devices, as this was the most frequently used device and because daily RHR and sleep algorithms vary across devices. Participants’ baseline mean and standard deviation (STD) for RHR, steps, and sleep were calculated using all data collected more than 7 days before symptom onset. Participants had to have >30 days of baseline data to be included in the sensor comparison. A daily *z*-score was calculated using the following equation: *z*-score for RHR = (daily RHR− mean baseline RHR)/baseline STD RHR. The mean and 95% confidence interval (CI) of the participant’s daily *z*-score for RHR, steps, and sleep were calculated and compared across different conditions.

## Supplementary information

Supplemental Material

## Data Availability

All interested investigators will be allowed access to the deidentified analysis dataset if they register with Scripps Research’s institutional review board and pledge not to reidentify individuals or share the data with a third party. All data inquiries should be addressed to Jay Pandit (jpandit@scripps.edu). The study protocol, informed consent form, and programming code are also available upon request to the corresponding author.

## Code availability

The programming code is also available upon request to the corresponding author.
