# Supplementary material for: Long-term changes in wearable sensor data in people with and without Long Covid
Source: NPJ Digit Med. 2024 Sep 13;7:246. doi: 10.1038/s41746-024-01238-x (PMC11399345; doi:10.1038/s41746-024-01238-x)
Supplement: Supplementary file 1 — Supplemental Material [file 41746_2024_1238_MOESM1_ESM.pdf]

SUPPLEMENT:

Long-term Changes in Wearable Sensor Data in People with and without Long Covid

Jennifer M. Radin[1], Julia Moore Vogel[1], Felipe Delgado[1], Erin Coughlin[1], Matteo Gadaleta[1], Jay A. Pandit[1], Steven R. Steinhubl[1,2]

1. Scripps Research Translational Institute, La Jolla, CA 92037
2. Purdue University, Weldon School of Biomedical Engineering, West Lafayette, IN 47907 USA

Supplementary Figure 1. Number of participants contributing daily sensor data by metric over time since symptom onset (days -7 to 365) (a) Resting Heart Rate (RHR), (b) steps, (c) sleep quantity

a.

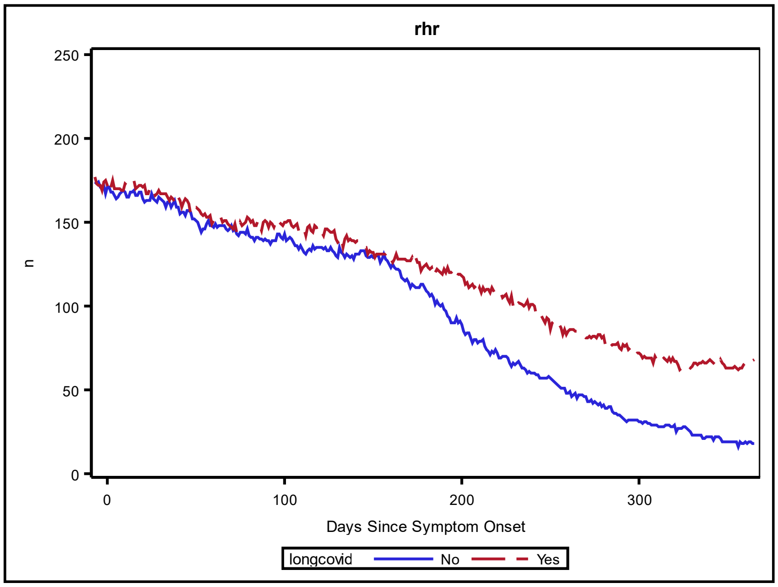

b.

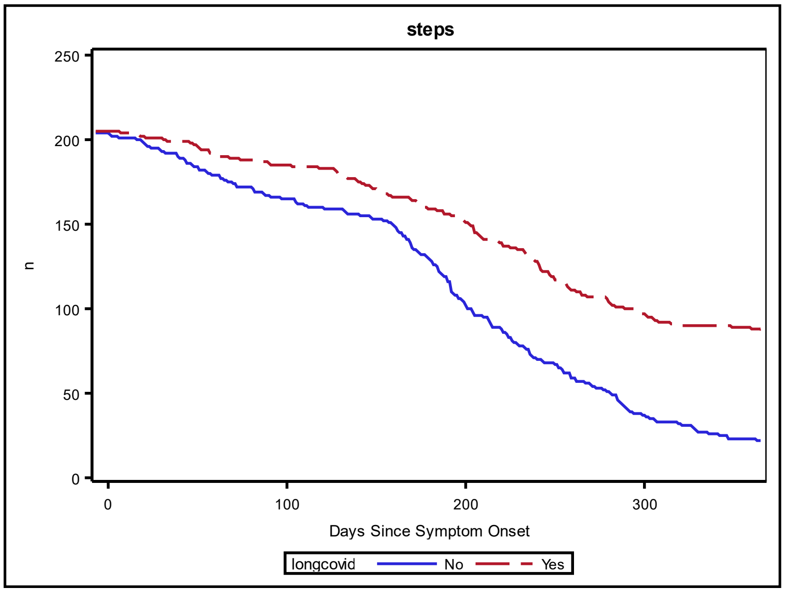

c.

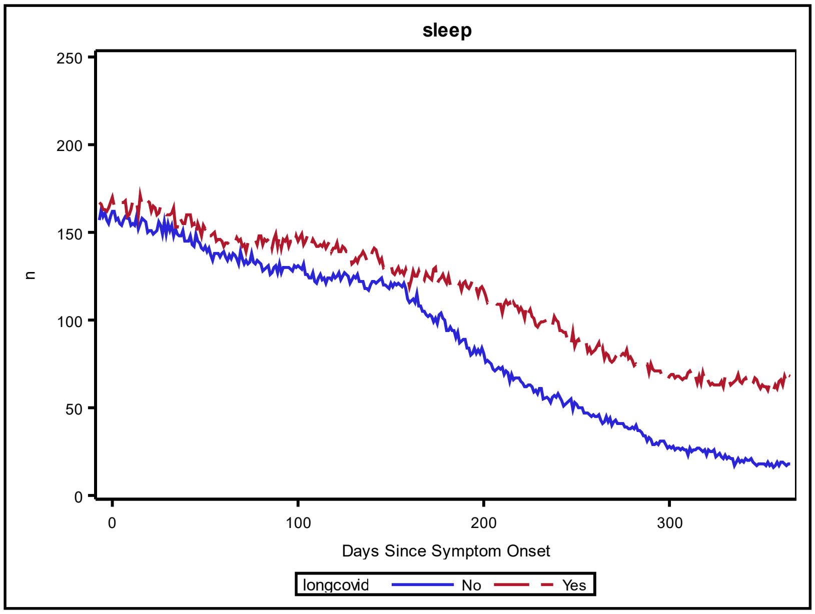

sleep
250
200
150
100
50
0
n
0
100
200
300
Days Since Symptom Onset
longcovid
No
Yes
